# Long-term survival following chemoradiotherapy for postoperative recurrence of hilar cholangiocarcinoma

**DOI:** 10.1007/s12328-026-02280-w

**Published:** 2026-01-31

**Authors:** Kazuki Asakura, Takahiro Einama, Kazuki Kobayashi, Hanae Shinada, Naoto Yonamine, Takazumi Tsunenari, Mikiya Takao, Saburo Matsubara, Kazuyuki Oyama, Yoji Kishi

**Affiliations:** 1 Department of Surgery, National Defense Medical College, 3-2 Namiki, Tokorozawa, Saitama, 359-8513 Japan; 2 Department of Gastroenterology and Hepatology, Saitama Medical Center, Saitama Medical University, 1981 Kamoda, Kawagoe, Saitama, 350-8550 Japan; 3 Department of Radiology, Shin-Yamanote Hospital, 3-6-1 Suwacho, Higashimurayama, Tokyo 189-0021 Japan

**Keywords:** Cholangiocarcinoma, Recurrence, Chemoradiotherapy, Oligometastasis

## Abstract

Standard treatment for metastatic biliary tract cancer is systemic chemotherapy, not local therapy, such as surgery or radiation therapy. This is because long-term survival is not expected with local therapy. We present the case of a 49-year-old man who underwent extended right hepatectomy with extrahepatic bile duct resection for hilar cholangiocarcinoma. Postoperative adjuvant chemotherapy with oral S-1 for six months was completed. At 20 months post-operation, para-aortic lymphadenopathy was detected, and endoscopic ultrasound-guided biopsy confirmed lymph node recurrence. Gemcitabine and cisplatin were administered for six months, resulting in partial tumor regression. As no other lesions were identified, radiotherapy with a total dose of 55 Gy in 22 fractions was performed. At 37 months following the completion of radiotherapy, the patient remained disease-free based on imaging. This case suggests that the addition of local radiotherapy to systemic chemotherapy may be beneficial for patients with oligometastatic recurrence of hilar cholangiocarcinoma.

## Introduction

Chemotherapy is the standard treatment for metastatic and recurrent biliary tract cancer (BTC) [1]. However, multimodal treatment, including local treatment such as surgical resection and radiotherapy after systemic chemotherapy, have been reported to be effective in patients with oligometastatic recurrence [2, 3].

In BTC, radiotherapy is used mainly for palliative treatment of bone metastases, but its efficacy for locoregional treatment of lymph node metastasis in hilar cholangiocarcinoma remains unclear, and current clinical guidelines provide no clear recommendations [1].

Herein, we report a patient who achieved long-term survival following chemoradiotherapy for postoperative lymph node metastasis of hilar cholangiocarcinoma.

## Case presentation

**Fig. 1:**
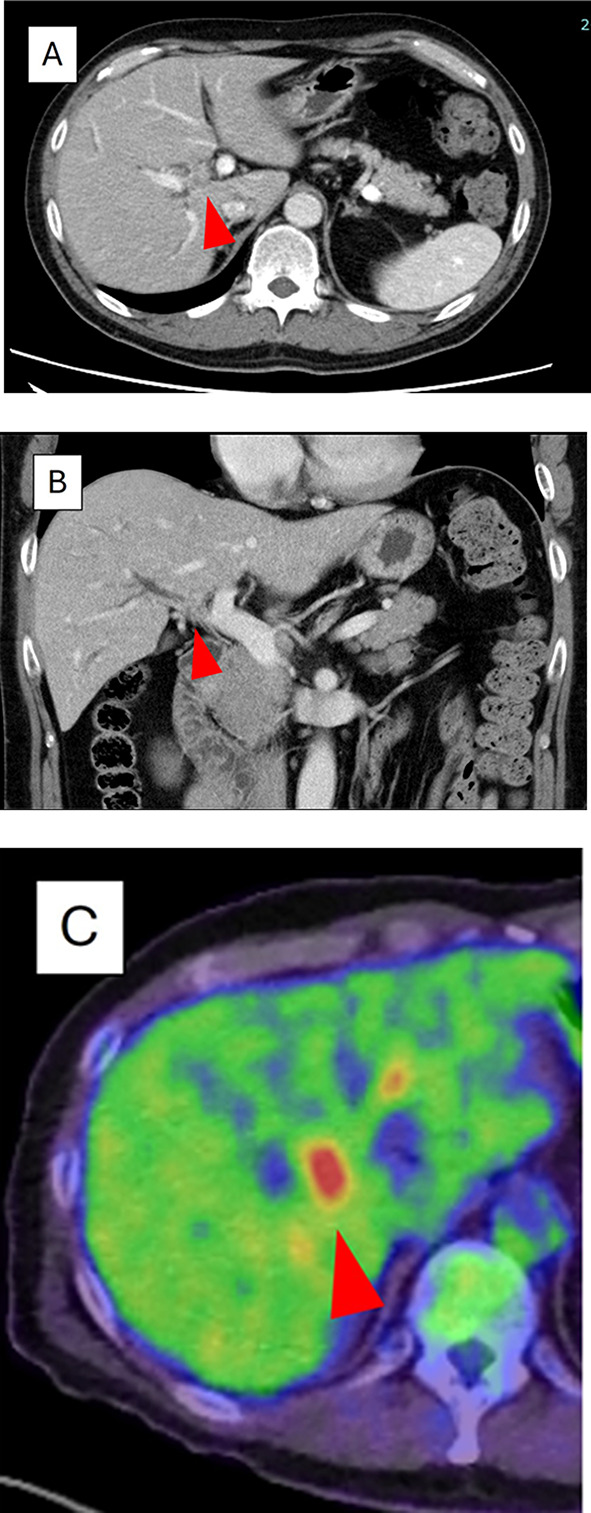
Preoperative imaging. **A** Contrast-enhanced abdominal CT (axial view) showing a contrast-enhanced mass at the hepatic hilum (arrowhead). **B** Coronal view of the same lesion as in (**A**) (arrowhead). (**C**) FDG-PET demonstrating increased uptake in that region (arrowhead)

A 49-year-old man with no significant past medical history or symptoms was diagnosed with hepatic dysfunction during a routine medical check-up. CT revealed a mass in the hepatic hilum, and subsequent workup confirmed the diagnosis of hilar cholangiocarcinoma (Fig. 1a–c). The patient underwent extended right hepatectomy with extrahepatic bile duct resection. Pathological examination led to a diagnosis of well-differentiated adenocarcinoma, stage IIIc (T2bN1M0) based on the 8th Union for International Cancer Control staging.

**Fig. 2:**
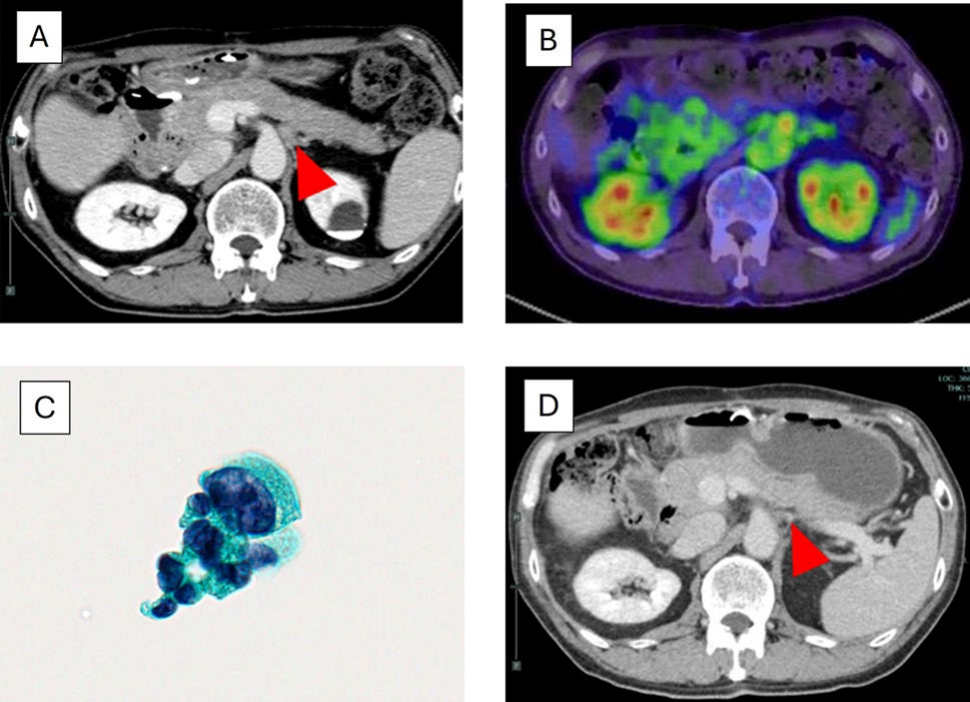
Imaging series after tumor recurrence. **A** At 20 months after surgery, contrast-enhanced abdominal CT (axial view) revealed increased soft-tissue density around the aorta (arrowhead). **B** FDG-PET showed increased uptake in that region. **C** Endoscopic ultrasound-guided biopsy was performed at the same site, and the cytological examination revealed atypical cells, leading to a Class V diagnosis. **D** At 28 months postoperatively, contrast-enhanced CT showed shrinkage of the lesion (arrowhead)

**Fig. 3:**
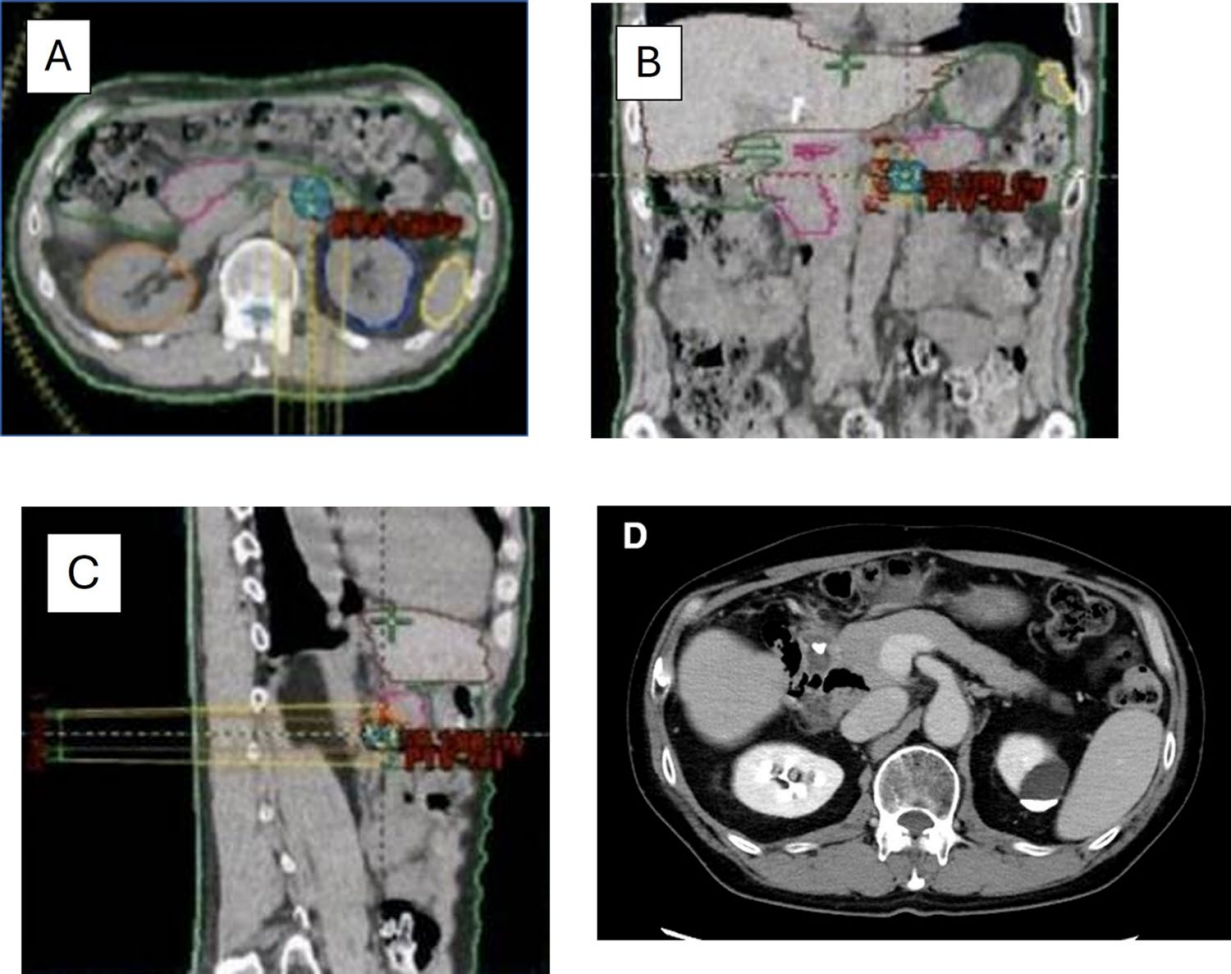
Radiotherapy planning CT. **A** Axial view, **B** coronal view, **C** sagittal view and **D** CT (axial view) at 50 months after surgery. There was no lymph node swelling

Adjuvant chemotherapy with S-1 (120 mg/day: twice a day for 2 weeks with subsequent withdrawal for 1 week) was administered for six months [4], followed by routine surveillance. At 20 months postoperatively, CT revealed para-aortic lymphadenopathy, and endoscopic ultrasound-guided biopsy confirmed nodal recurrence (Fig. 2a–c). CEA and CA19-9 were not elevated in this patient during all treatment periods. We performed six cycles of gemcitabine and cisplatin combination therapy (GC therapy: gemcitabine, 1000 mg/m^2^; and cisplatin, 25 mg/m^2^). After that, CT confirmed size reduction of lymph node metastases and no evidence of new lesions (Fig. 2d). The subsequent treatment strategy was determined following discussion in a multidisciplinary team conference. As there was a possibility that the shrunken lymph nodes might not be detected during surgery, the discussion reached our multidisciplinary consensus as follows: 1. Local therapy after systemic treatment for oligometastasis is challenging but acceptable if the goal is to aim for a cure. 2. Radiation therapy is preferred because there is a possibility that the shrunken lymph nodes might not be detected during surgery. Radiotherapy with a total of 55 Gy in 22 fractions was selected, radiotherapy procedure was Intensity Modulated Radiation Therapy (IMRT), Gross Tumor Volume (GTV) was 0.8cm^3^ (mean 55.594 Gy, 57.217 Gy–74.054 Gy), Clinical Target Volume (CTV) was 2.3cm^3^ (mean 55.137 Gy, 58.436 Gy–51.157 Gy) and Planning Target Volume (PTV) was 10.3cm^3^ (mean 49.931 Gy, 59.188 Gy-35.778 Gy). The patient had no acute and late toxicity (Fig. 3a-c). We prescribed S-1 for six months, including the period of radiation therapy. A complete response was confirmed by CT at 33 months postoperatively and the patient remains alive and disease-free 58 months after surgery (Fig. 3d).

## Discussion

The recurrence rate after curative resection of hilar cholangiocarcinoma is approximately 60% [5, 6]. We typically perform systemic chemotherapies for recurrence, but the survival period after recurrence is an average of about one year [7]. In the present case, the partial tumor response to GC therapy enabled us to add local radiotherapy, and a complete clinical response and long-term survival without further chemotherapy could be achieved.

GC therapy has been the standard first-line regimen for advanced biliary tract cancers since the ABC-02 trial [7]. More recently, combination therapies such as GC + S-1 [8], GC + Durvalumab [9], and GC + Pembrolizumab [10] regimens have also demonstrated efficacy. This case occurred in the 2010s, and we selected GC therapy as it had the highest response rate at the time. Furthermore, we are now able to perform Comprehensive Genomic Profiling (CGP) using surgical specimens for recurrent cases and select anticancer drugs accordingly. Since GC therapy was effective in this case, there is a possibility that the patient may have had a BRCA mutation [11]. For limited local recurrence, surgical resection is recommended when feasible [12], and radiotherapy has shown potential as an alternative. Marino et al. introduced the concept of oligometastatic recurrence, suggesting that patients with up to three lesions in a single organ may benefit from aggressive local treatment [2]. In patients with oligometastatic biliary cancer recurrence, stereotactic body radiotherapy (SBRT) after chemotherapy yielded a median survival time (MST) of 13.7 months [13]. Although this case involved radiation therapy, we performed S-1 administration for 6 months as an adjuvant therapy in accordance with the ASCOT trial [4].

A literature search of PubMed was conducted with the aim of identifying the most recent English reports. In the search strategy, search terms used were: "bile duct cancer," "recurrent," "chemotherapy," and "radiation." We identified four patients, including ours, in whom a complete response was achieved with chemoradiotherapy (Table 1) [14–16]. There are only four case reports from Japan. These abstracts are available in English, and we provided case details in Table 1. All showed oligometastatic recurrence, and radiotherapy was typically chosen due to the difficulty of achieving local control with systemic chemotherapy. In our case, the metastatic lesion was biopsied and distant metastasis was confirmed pathologically. These findings suggest that multidisciplinary treatment for oligometastasis of bile duct cancer might result in long-term survival. Conversion therapy for biliary tract cancer was not common at the time, so our discussion was based on that for gastric cancer. The best timing for the operation is generally when the tumor displays the best response to chemotherapy. Specifically, it stated that it was the best timing for the removal of the tumor to be when a CR or PR response is detected during the performance of 4–6 cycles of S-1/Cisplatin or S-1/docetaxel regimens [17]. So, we decided the timing of locoregional therapy after chemotherapy with sustained PR and a similar duration.

**Table 1:** Biliary tract cancer patients who achieved complete response with chemoradiotherapy

Author	Year	Age	Sex	Diagnosis	Recurrent site	Chemotherapy regime (months)	Radiotherapy	Other treatment after radiotherapy	Prognosis (months)
Hashimoto et al	2019	62	M	GB Ca	Lymph node	Gemcitabine + Cisplatin (4 months) Gemcitabine + S-1 (2 months)	50.4 Gy	Resection	Alive (5 months)
Ishida et al	2020	70	F	Hilar Ca	Lung	Gemcitabine + Cisplatin (4 months)	50G y	Resection	Alive (28 months)
Sakai et al	2023	80’s	M	CCC	Resection site	Gemcitabine + Cisplatin + S-1 (3 months) Gemcitabine + Cisplatin (2 month) Gemcitabine + S-1 (1 month) Gemcitabine (6 months) S-1 (15 months)	50 Gy	None	Alive (12 months)
Our case	2025	49	M	Hilar Ca	Lymph node	Gemcitabine + Cisplatin (6 months)	55 Gy	None	Alive (50 months)

GB Ca, gallbladder carcinoma; Hilar Ca., hilar cholangiocarcinoma; CCC, cholangiocellular carcinoma

Usually, the primary goals of radiotherapy include palliation, maintenance of stent patency, relief from jaundice, and pain management. Shinchi et al. reported that survival of patients with unresectable hilar cholangiocarcinoma was more favorable in those who received radiotherapy than in those who did not (10.6 vs. 4.4 months, respectively) [18]. Moreover, chemoradiotherapy for lymph node metastasis resulted in a better overall survival than radiotherapy alone (31 vs. 13 months, respectively, *p* < 0.001) [19]. The combination of radiation therapy and anticancer drugs is called chemoradiotherapy, and is based on local cooperation, the mutual effects of radiation and drugs improve local efficacy, and spatial cooperation, chemotherapy controls invisible distant metastases in areas not exposed to radiation. In biliary tract cancer, Kobayashi et al. reported that combination therapy with gemcitabine and radiation therapy might have the potential to improve recurrence-free survival and overall survival [20]. In this case, chemotherapy was administered first, like induction therapy, followed by concurrent chemoradiotherapy of S-1. These treatments might have improved the cure rate. Despite its potential effect of prolonging survival, radiotherapy has not yet been standardized; particularly, the appropriate timing, dosage, and indications remain unclear. To establish standardized treatment protocols including local therapy, large-scale clinical studies are necessary.

"Long-term survival" typically refers to surviving for five years or more after a cancer diagnosis. It is used as an indicator of the percentage of patients who survive without cancer recurrence a certain period (generally five years) after treatment has ended, and based on past data, it has been considered a benchmark for "cure." In this case, although it has only been about three years since local treatment, it has been almost five years since the initial resection, so we have considered the patient to be a long-term survivor.

## Conclusion

We reported a patient with hilar cholangiocarcinoma and postoperative oligometastatic recurrence successfully treated with chemoradiotherapy, resulting in long-term recurrence-free survival. Radiotherapy may be a viable treatment option in selected patients with limited metastatic disease following chemotherapy.

## References

[1] Vogel A, Bridgewater J, Edeline J, et al. Biliary tract cancer: ESMO clinical practice guideline for diagnosis, treatment and follow-up. Ann Oncol. 2023;34:127–40. 36372281 10.1016/j.annonc.2022.10.506

[2] Morino K, Seo S, Yoh T, et al. Proposed definition for oligometastatic recurrence in biliary tract cancer based on results of locoregional treatment: a propensity-score-stratified analysis. Ann Surg Oncol. 2020;27:1908–17. 31939034 10.1245/s10434-020-08207-0

[3] Sota Y, Einama T, Kobayashibayashi K, et al. Recurrent cholangiocarcinoma with long-term survival by multimodal treatment: a case report. Mol Clin Oncol. 2021;14:72. 33732458 10.3892/mco.2021.2234 PMC7907798

[4] Nakachi K, Ikeda M, Konishi M, et al. Adjuvant S-1 compared with observation in resected biliary tract cancer (JCOG1202, ASCOT): a multicentre, open-label, randomised, controlled, phase 3 trial. Lancet. 2023;401:195–203. 36681415 10.1016/S0140-6736(22)02038-4

[5] Groot Koerkamp B, Wiggers JK, Allen PJ, et al. Recurrence rate and pattern of Perihilar cholangiocarcinoma after curative intent resection. J Am Coll Surg. 2015;221:1041–9. 26454735 10.1016/j.jamcollsurg.2015.09.005 PMC4736142

[6] Komaya K, Ebata T, Yokoyama Y, et al. Recurrence after curative-intent resection of perihilar cholangiocarcinoma: analysis of a large cohort with a close postoperative follow-up approach. Surgery. 2018;163:732–8. 29336813 10.1016/j.surg.2017.08.011

[7] Valle J, Wasan H, Palmer DH, et al. Cisplatin plus gemcitabine versus gemcitabine for biliary tract cancer. N Engl J Med. 2010;362:1273–81. 20375404 10.1056/NEJMoa0908721

[8] Ioka T, Kanai M, Kobayashi S, et al. Randomized phase III study of gemcitabine, cisplatin plus S-1 versus gemcitabine, cisplatin for advanced biliary tract cancer (KHBO1401- MITSUBA). J Hepatobiliary Pancreat Sci. 2023;30:102–10. 35900311 10.1002/jhbp.1219 PMC10086809

[9] Oh DY, He AR, Bouattour M, et al. Durvalumab or placebo plus gemcitabine and cisplatin in participants with advanced biliary tract cancer (TOPAZ-1): updated overall survival from a randomised phase 3 study. Lancet Gastroenterol Hepatol. 2024;9:694–704. 38823398 10.1016/S2468-1253(24)00095-5

[10] Kelley RK, Ueno M, Yoo C, et al. Pembrolizumab in combination with gemcitabine and cisplatin compared with gemcitabine and cisplatin alone for patients with advanced biliary tract cancer (KEYNOTE-966): a randomised, double-blind, placebo-controlled, phase 3 trial. Lancet. 2023;401:1853–65. 37075781 10.1016/S0140-6736(23)00727-4

[11] Hewitt DB, Aziz H, Brown ZJ, et al. Role of genetic testing in hepatic, pancreatic, and biliary cancers. Surg Oncol. 2022;44:101844. 36116416 10.1016/j.suronc.2022.101844

[12] Noji T, Tsuchikawa T, Mizota T, et al. Surgery for recurrent biliary carcinoma: results for 27 recurrent cases. World J Surg Oncol. 2015;13:82. 25884694 10.1186/s12957-015-0507-8 PMC4350290

[13] Franzese C, Bonu ML, Comito T, et al. Stereotactic body radiotherapy in the management of oligometastatic and recurrent biliary tract cancer: single-institution analysis of outcome and toxicity. J Cancer Res Clin Oncol. 2020;146:2289–97. 32524292 10.1007/s00432-020-03285-9 PMC11804481

[14] Hashimoto Y, Kishimoto T, Murotani M, et al. A case of long-term survival of liver metastasis from biliary carcinoma after pancreaticoduodenectomy treated by multimodal therapy. Gan Kagaku Ryoho. 2019;46:1987–9. 32157035

[15] Ishida H, Seyama Y, Ome Y, et al. A case of pulmonary metastasis from hilar cholangiocarcinoma treated by stereotactic body radiotherapy. Gan Kagaku Ryoho. 2020;47:340–2. 32381982

[16] Sakai K, Gotoh K, Toshiyama R, et al. A case of long-term survival with multidisciplinary treatment for postoperative local recurrence of intrahepatic cholangiocarcinoma. Gan Kagaku Ryoho. 2023;50:1795–7. 38303210

[17] Yoshida K, Yamaguchi K, Okumura N, et al. Is conversion therapy possible in stage IV gastric cancer: the proposal of new biological categories of classification. Gastric Cancer. 2016;19:329–38. 26643880 10.1007/s10120-015-0575-z PMC4824831

[18] Shinchi H, Takao S, Nishida H, et al. Length and quality of survival following external beam radiotherapy combined with expandable metallic stent for unresectable hilar cholangiocarcinoma. J Surg Oncol. 2000;75:89–94. 11064386 10.1002/1096-9098(200010)75:2<89::aid-jso3>3.0.co;2-v

[19] Yoshioka Y, Ogawa K, Oikawa H, et al. Factors influencing survival outcome for radiotherapy for biliary tract cancer: a multicenter retrospective study. Radiother Oncol. 2014;110:546–52. 24560766 10.1016/j.radonc.2014.01.003

[20] Kobayashi S, Tomokuni A, Gotoh K, et al. A retrospective analysis of the clinical effects of neoadjuvant combination therapy with full-dose gemcitabine and radiation therapy in patients with biliary tract cancer. Eur J Surg Oncol. 2017;43:763–71. 28100416 10.1016/j.ejso.2016.12.008

